# LINC01123 promotes immune escape by sponging miR-214-3p to regulate *B7–H3* in head and neck squamous-cell carcinoma

**DOI:** 10.1038/s41419-022-04542-0

**Published:** 2022-02-03

**Authors:** Huan Li, Zihui Yang, Xiangming Yang, Fengrui Zhang, Jun Wang, Zhongming Wu, Chaojie Wanyan, Qingzhe Meng, Wanpeng Gao, Xinjie Yang, Jianhua Wei

**Affiliations:** State Key Laboratory of Military Stomatology and National Clinical Research Center for Oral Diseases, and Shaanxi Clinical Research Center for Oral Diseases, Department of Oral and Maxillofacial Surgery, School of Stomatology, Fourth Military Medical University, Xi’an, 710032 China

**Keywords:** Head and neck cancer, Immune evasion

## Abstract

Numerous studies have shown that long noncoding RNAs (LncRNAs) are involved in the development and immune escape of head and neck squamous-cell carcinoma (HNSCC). However, the specific regulatory mechanisms by which LINC01123 regulates HNSCC and its correlation with immunity remain unclear. Therefore, this study’s primary purpose was to explore the mechanisms by which LINC01123 regulates the immune escape and progression of HNSCC. This study confirmed that LINC01123 is competitively bound to miR-214-3p, and miR-214-3p specifically targets *B7–H3*. The effects of LINC01123, *B7–H3*, and miR-214-3p on tumor progression, CD8^+^T-cell-mediated immune response, and the tumorigenicity of HNSCC in vitro and in vivo were examined through the downregulation or upregulation of LINC01123, *B7–H3*, and miR-214-3p. Our results indicated that LINC01123 and *B7–H3* were highly expressed in HNSCC and are associated with poor prognosis in patients. Notably, overexpression of LINC01123 or *B7–H3* or downregulation of miR-214-3p inhibited the function of CD8^+^T cells and promoted the progression of HNSCC. Therefore, LINC01123 acts as a miR-214-3p sponge to inhibit the activation of CD8^+^T cells and promote the progression of HNSCC by upregulating *B7–H3*.

## Introduction

Head and neck squamous-cell carcinoma (HNSCC), an immunosuppressive tumor with high malignancy, is the sixth most common cancer in the world [1, 2]. The current conventional treatments for HNSCC are surgery, adjuvant radiotherapy, and chemotherapy. Although treatment technologies and concepts are constantly updated [3], patients’ 5-year survival rates are still poor [4]. Immunotherapy research has recently achieved a great breakthrough, but immune-checkpoint inhibitors are not effective in many patients. Clinically, only 20% of patients actually respond to these cancer immunotherapies [5]. The low response rate is mainly a result of the complex immune escape mechanisms of HNSCC. Therefore, a better understanding of the biological characteristics of HNSCC cell immune-escape is key to improving patients’ prognoses.

Long noncoding RNA (LncRNA) are functional RNA molecules with lengths greater than 200 nucleotides that do not encode proteins and are closely related to the occurrence and development of many tumors [6, 7]. A recent study suggested that LncRNA can regulate the expression of critical molecules by tumor cells, allowing them to evade and impair-immune system activity [8]. For example, LINC00473 sponges miR-195-5p to affect pancreatic cancer development by regulating PD-L1 and inhibiting the activation of CD8^+^T cells [9]. Moreover, analysis of immune-escape-related LncRNA can even predict the response to anti-PD-1 immunotherapy [10]. Therefore, the emerging role of LncRNA in immunotherapy has become a hot topic of research [11]. LINC01123 can accelerate hepatocellular carcinoma metastasis by targeting the miR-34a-5p/TUFT1 axis [12], and it is highly expressed in non-small-cell lung cancer [13], colon cancer [14], and endometrial cancer [15]. LINC01123 is related to cancer prognosis and regulates cell proliferation, invasion, and metastasis. Furthermore, researchers have developed a powerful 4-LncRNA prognostic signature for HNSCC, including RP11-366H4.1, LINC01123, RP11-110I1.14, and CTD-2506J14.1, which are important prognostic factors independent of multiple clinicopathological parameters [16]. These results indicate that LINC01123 is closely related to the prognosis of HNSCC, but the specific regulatory mechanisms of LINC01123 in HNSCC and its correlation with immunity are still unclear.

*B7–H3* (*CD276*), one of the B7 superfamily molecules [17], is highly expressed in a variety of tumors, leading to poor patient prognoses [18], and it can participate in lymphoma immune escape by regulating cytotoxic lymphocyte activation [19]. Recent studies showed that *B7–H3* blockade is a potential therapeutic strategy for treating patients with HNSCC; [20] Therefore, *B7–H3* may become a novel target for immunotherapy against cancer. Considering that *B7–H3* is involved in the immune-escape mechanism of HNSCC, we questioned if LINC01123 can regulate *B7–H3* expression, participate in the immune-escape process, and ultimately, contribute to the development of HNSCC.

In this study, we found that LINC01123 was significantly upregulated in samples from HNSCC patients. The transfection of LINC01123 sh-RNA into HNSCC cells inhibited the expression of *B7–H3* and enhanced the killing effect of CD8^+^T cells. Through bioinformatics analysis, we constructed a new competing endogenous RNA (ceRNA) network [21] (LINC01123/miR-214-3p/*B7–H3*). We also conducted a literature review and found that miR-214-3p was abnormally expressed in various malignant tumors and was closely related to the progression of malignant tumors [22, 23]. Our results demonstrated that the abnormally high levels of LINC01123 functioned as ceRNAs that sponged miR-214-3p, which led to the upregulation of *B7–H3*, thereby inhibiting the activation of CD8^+^T cells and promoting the progression of HNSCC.

## Materials and methods

### Cell lines and cell culture

An HNSCC cell line (SCC-9, SCC-15) and 293 T cells were initially purchased from the American Type Culture Collection (ATCC, USA). CAL-27, SCC-25, and Fadu cell lines were obtained from the Peking University School of Stomatology (Beijing, China). The SCC-7 cell line was obtained from the Fudan University (Shanghai, China). The human oral epithelial cell (HOEC) was obtained from the China Center for Type Culture Collection (CCTCC, China). Anonymized human blood samples were purchased from the Blood Transfusion Department of Xijing Hospital (Xian, China), and whole peripheral blood mononuclear cells (PBMCs) were isolated by density-gradient centrifugation using Ficoll Pacque Plus (GE Healthcare, Rydalmere, Australia). We isolated CD8^+^T cells from PBMCs using CD8 MicroBeads (#130-045-201, Miltenyi Biotec, Gladbach, Germany). CD8^+^T cells were then stimulated with the T cell Activation/Expansion kit (#130-091-441, Miltenyi Biotec). All cell lines were cultured in RPMI-1640 medium containing 10% fetal bovine serum at 37 °C in 5% CO_2_.

### LncRNA-seq sample preparation and sequencing

Total RNA was extracted from tumor cells (CAL-27, Fadu, SCC-9, and SCC-15) and HOEC cells using Trizol (Invitrogen, Carlsbad, CA, USA). The concentration and purity of total RNA were detected to make sure the samples could be used for LncRNA-Seq using a NanoPhotometer spectrophotometer (Implen, CA, USA). RNA integrity and quantity were finally measured using an RNA Nano 6000 Assay Kit for the Bioanalyzer 2100 system (Agilent Technologies, CA, USA). After library preparation, the samples were subjected to Illumina sequencing (Novogene Co., Ltd., Beijing, China).

### FISH assay

HNSCC cells (CAL-27, SCC-15, SCC-9, and Fadu) were seeded onto slides and cultured until the confluency reached 40–50%. The cells were fixed with 4% paraformaldehyde, washed with PBS twice, treated with 20 µg/ml of Proteinase K, prehybridized with prehybridization buffer at 37 °C for 1 h, and hybridized with a biotin-labeled LINC01123 probe (Servicebio Technology Co., Ltd., Wuhan, China) overnight in the absence of light at 37 °C. The cells were subsequently washed with washing buffers I (2 × SSC), II (1 × SSC), and III (0.5 × SSC) (10 min each time). Nuclei were stained with DAPI for 8 min at room temperature. The images were observed and collated under a fluorescence microscope (Nikon, Japan).

### Bioinformatic analysis

The differentially expressed genes (DEGs) of CAL-27, Fadu, SCC-9, SCC-15, and HOEC were analyzed by sequencing. EdgeR package (3.2.4) was used to analyze the significance of DEGs. *P* < 0.05 and | log2 (fold change)| >1 were set as the threshold for significantly differential expression. The hierarchical clustering method was adopted, and the results were represented by a heatmap. Venn diagram showed the upregulated LncRNAs of four HNSCC cell lines (CAL-27, Fadu, SCC-9, and SCC-15) compared with HOEC. We obtained HNSCC LncRNA sequencing data from TCGA public database (519 tumor cases and 44 normal samples). Moreover, patient-survival analyses of LINC01123 and *B7–H3* were also conducted using these data. The potential miRNA link between LINC01123 and *B7–H3* was predicted using LncBase [24] and Targetscan [25] (http://www.microrna.gr/LncBase, http://www.targetscan.org).

### Dual-luciferase-reporter gene assay

We applied the dual-luciferase-reporter gene assay to confirm that LINC01123 and *B7–H3* were both direct targets of miR-214-3p. The wild-type (WT) and mutated (MUT) sequences of the 3′UTR of LINC01123 and *B7–H3* mRNA were cloned into pmirGLO plasmids (GenePharma Co., Ltd., Shanghai, China). Luciferase constructs were cotransfected into 293 T cells together with miR-214-3p mimic or normal-control (NC) mimic, and the empty pmirGLO vector was used for the control group. The miR-214-3p inhibitor sequence was repeated twice and inserted into the pmirGLO plasmids as the positive-control (PC) group. After 48 h of transfection, the cells were harvested and analyzed with the dual-luciferase-reporter assay system (Promega, Madison, WI, USA) and the Infinite M1000 (Tecan, Morrisville, NC, USA) fluorescent-plate reader.

### Cell grouping

All cells in the logarithmic growth phase were divided into nine groups: the blank group (cells transfected without any sequence); the NC group (cells transfected with normal-control plasmids), the sh-LINC01123 group (cells transfected with sh-LINC01123), the OE-LINC01123 group (cells transfected with LINC01123 overexpression plasmid), the miR-214-3p mimic group (cells transfected with miR-214-3p mimic), the miR-214-3p inhibitor group (cells transfected with miR-214-3p inhibitor), the sh-*B7–H3* group (cells transfected with sh-*B7–H3*), the sh-LINC01123 + inhibitor–NC group (cells cotransfected with sh-LINC01123 and inhibitor normal-control plasmids), and the sh-LINC01123 + miR-214-3p inhibitor group (cells cotransfected with sh-LINC01123 and miR-214-3p inhibitor plasmids).

### Plasmid transfection

HNSCC cells were inoculated into 24-well plates at a density of 2 × 10^5^ cells/well and transfected using Lipofectamine 2000 (Invitrogen) when the cell confluence reached 60–70%. Subsequently, 5 μg of target plasmid and 12 μL of Lipofectamine 2000 were diluted independently with 250 μL of serum-free Opti-MEM medium (Invitrogen). The diluted plasmid was added into the diluted Lipofectamine 2000 Reagent at a 1:1 ratio and incubated for 5 min at room temperature. The plasmid–lipid complex was then added to the culture wells for incubation at 37 °C in 5% CO_2_. Complete culture medium (10% FBS RPMI-1640) was replaced after 12 h, the cells were collected after 48 h, and the transfected cells were analyzed.

### qRT-PCR

The present research was approved by the Medical Research Ethics Committee of the Fourth Military Medical University, and the informed consent was obtained from all the patients. HNSCC tissues (Num=19) and paracancerous tissues (Num=19) were collected, and none of the patients received radiotherapy or chemotherapy before surgery. The human population, sample size, clinical characteristics, and HPV status were detailed in the supplementary materials. Total RNA was extracted from human tissues and cell lines (CAL-27, Fadu, SCC-25, SCC-15, SCC-9, and HOEC) using the Takara MiniBEST Universal RNA Extraction Kit (Takara Bio, Inc., Otsu, Japan) and reverse-transcribed into cDNA according to the instructions of the PrimeScript RT reagent kit (Takara, Japan) or TaqMan MicroRNA Reverse Transcription Kit (Applied Biosystems, Carlsbad, CA, USA). The qRT-PCR was performed using the SYBR Premix Ex Taq Kit (Takara) or miRcute miRNA qPCR Detection Kit (TIANGEN, Beijing, China). U6 was used as the internal reference for miR-214-3p, and β-actin served as the internal control for LINC01123 and *B7–H3*. The primers were synthesized by Sangon Biotechnology Inc. (Shanghai, China) (Table 1). The relative expression level of the target genes was calculated with the 2^−^^ΔΔCt^ method.

**Table 1:** The list of primers and probes.

Gene	Primer	Sequence(5′−3′)
*Primers for qRT-PCR*		
LINC01123	Forward	CAGCAGCAGCAGTGAGGAAGC
	Reverse	CGACCGAGGTGACAACGATGAC
miR-214-3p	Forward	GCGACAGCAGGCACAGACA
	Reverse	AGTGCAGGGTCCGAGGTATT
*B7–H3*	Forward	CTGACAGATACCAAACAGCTGG
	Reverse	CGAAATCCCGGATGCTCA
U6	Forward	CTCGCTTCGGCAGCACA
	Reverse	CTCAACTGGTGTCGTGGA
β-actin	Forward	TGACGTGGACATCCGCAAAG
	Reverse	CTGGAAGGTGGACAGCGAGG
*Primers for stem-loop RT-PCR of miRNAs*		
U6	RT Primer	AACGCTTCACGAATTTGCGT
miR-214-3p	RT Primer	GTCGTATCCAGTGCAGGGTCCGAGGTATTCGCACTGGATACGACACTGCC
Sequences of probes for RNA FISH		
LINC01123 5’-DIG- CCCTTGCATGGTACACTTGAAATAGATGGGCTGGA-DIG-3’		
sh-RNAs target sequence		
sh-*B7–H3*	Homo sapiens	GCAGCTGACAGATACCAAACA
sh-*B7–H3*	mouse	GGAAGTCCAGGTCTCTGAAGA

### Western blot analysis

Total protein was extracted from each sample, separated on 10% SDS-PAGE, and transferred to PVDF membranes. The membranes were then blocked with 5% skimmed milk powder in TBST for 2 h and immunoblotted with anti-*B7–H3* (1:500, GTX34211, GeneTex, Inc., Texas, USA) and anti-β-actin (1:1000, GTX109639, GeneTex, Inc.) at 4 °C overnight. Afterward, the membrane was incubated with goat anti-rabbit IgG H&L (HRP) (Abcam, Cambridge, UK, ab6721, 1:2000) at 37 °C for 2 h. The proteins were visualized using enhanced chemiluminescence reagent (Thermo, USA), and the images were processed using Quantity One 4.4.0 software.

### CCK8-proliferation assay

Nine groups of differently treated CAL-27 and SCC-15 cells were inoculated into 96-well plates in triplicate at a density of 2 × 10^3^ cells/well. The cells were incubated for 24, 48, and 72 h before 10 μl of Cell Counting Kit 8 (CCK8) (7sea Pharmatech Co., Ltd., Shanghai, China) was added to each well. After incubation for 1 h, the OD values were measured at 450 nm.

### Scratch wound healing assay

HNSCC cells (CAL-27 and SCC-15) were inoculated into 6-well plates at a density of 1 × 10^6^ cells/well. Scratch wound-healing assays were conducted 48 h after transfection. A scratch was made in each well with a sterile 200-μL pipette tip, and the wells were washed twice with serum-free RPMI-1640 to remove the detached cells. After 24 h of culture in complete medium, we captured images of the cells using an inverted light microscope (Olympus, Center Valley, PA).

### Migration and invasion assays

We seeded 5 × 10^4^ tumor cells (CAL-27 and SCC-15) into 200-μL serum-free RPMI-1640 in 8-μm transwell inserts (for migration assay, Corning, USA) or Matrigel-coated 8-μm transwell inserts (for invasion assay, Corning) of 24-well plates. RPMI-1640 medium containing 5% FBS was placed into the lower chamber. After 24 h of incubation, the cells in the upper chamber were removed with cotton swabs. Migrated cells were fixed using 4% formaldehyde and stained with 1% crystal violet. The number of cells that penetrated the membrane was counted in five random fields (400 × magnification).

### Flow-cytometry assay

Apoptosis detection and cell-cycle analysis were carried out using flow cytometry. For apoptosis detection, HNSCC cells (CAL-27 and SCC-15) were harvested with 0.25% trypsin without EDTA (Corning) 48 h after transfection. We employed the Annexin V-FITC/PI double-labeling apoptosis kit (BD Biosciences, San Diego, USA) to measure the apoptosis of tumor cells. The apoptosis rate was the sum of the early apoptosis rate (lower-right quadrant) and late apoptosis rate (upper-right quadrant). The cell cycle detection kit (4 A Biotech Co., Ltd., Beijing, China) was used to evaluate the cell-cycle-stage distribution. All experimental procedures were carried out according to the instructions.

Next, we analyzed the expression of TNF-α, IFN-γ, perforin, and granzyme B in CD8^+^T cells by flow cytometry after they were cocultured with different HNSCC cell treatment groups. After fixation and permeabilization with the transcription-factor buffer set (BD Biosciences, San Jose, CA, USA) for 40 min at 4 °C, human CD8^+^T cells were stained with anti-IFN-γ-PE (#12-7319-41, eBioscience, USA), anti-TNF-α-FITC (#11-7349-81, eBioscience), anti-granzyme B-PE (#12-8896-42, eBioscience), and anti-perforin-FITC (#11-9994-42, eBioscience) for 40 min at 4 °C while protected from light. The cells were washed twice and resuspended in flow-cytometry stain buffer for analysis. Flow cytometry experiments were performed using the flow cytometer Cytomics FC500 (Beckman Coulter Inc., Brea, CA, USA), and the results were analyzed using Flowjo software.

### ELISA

The supernatants of each group of cocultured CAL-27-CD8^+^T cells were collected to detect TNF-α, IFN-γ, perforin, and granzyme B expression using the corresponding human ELISA kits (Shanghai Westang Bio-Tech Co., Ltd., Shanghai, China) according to the instructions provided. To evaluate the effect of *B7–H3* on the immune activity of CD8^+^T cells, we detected the levels of TNF-α, IFN-γ, perforin, and granzyme B expression in the CD8^+^T-cell group supplemented with recombinant human *B7–H3* (#2318-B3, R&D Systems, Minneapolis, MN, USA).

ELISA was also used to detect the expression of TNF-α, IFN-γ, perforin, and granzyme B in the peripheral blood of tumor-bearing C3H mice. Fresh blood was collected from the mice by removing the eyeball. Blood samples were placed into sterile EP tubes, incubated at 37 °C for 1 h, and centrifuged at 3000 rpm for 10 min, and the supernatant collected. The OD value of each sample was read at 450 nm using the Denley Dragon Wellscan MK3 microplate reader (Thermo, Finland), and the concentrations were calculated based on a standardized curve.

### CCK8 cytotoxicity test of CD8^+^T cells

HNSCC cells subjected to different treatments were inoculated into 96-well plates at a density of 2 × 10^3^ cells/well. Three duplicate wells were set up for each group. Next, CD8^+^T cells were added at an effector to target ratio of 10:1. After 24 h of incubation, the supernatant was removed from each well. The wells were washed twice with PBS, and 10 μl of CCK8 and 90 μl of serum-free RPMI-1640 were added to each well. The OD value was measured at 450 nm after incubation for 1 h. Cytotoxicity% = (OD_control_ − OD_sample_)/OD_control_ × 100%.

### In vivo tumorigenicity assay

A total of 27 female C3H mice (aged 4–8 weeks, ~20 g) purchased from Vital River Laboratories (Beijing, China) were randomly assigned to nine groups with three mice in each group: the blank group, the NC group, the sh-LINC01123 group, the OE-LINC01123 group, the miR-214-3p mimic group, the miR-214-3p inhibitor group, the sh-*B7–H3* group, the sh-LINC01123 + inhibitor–NC group, and the sh-LINC01123 + miR-214-3p inhibitor group. HNSCC cells (SCC-7, 1 × 10^7^ cells) suspended in 0.2 mL of PBS were subcutaneously inoculated into the back of each C3H mouse. After approximately seven days, when the tumor volume reached 10 mm^3^, the tumors were treated by intratumoral injection of the liposome–DNA complex using a BD Precision Glide needle (BD, NJ, USA), and intratumoral multiple-point injection of complex was performed every three days. Tumor volume was measured weekly with a caliper and calculated using the standard formula: length × width^2^ × 0.5. After four weeks, the tumors were harvested and weighed. The C3H mice were then euthanized, and an image of each tumor was recorded. All mice were handled in accordance with protocols approved by the Committee on the Use of Live Animals in Teaching and Research of the Fourth Military Medical University.

### Immunofluorescence assay

The tumor samples were deparaffinized by two changes of xylene (15 min each), followed by dehydration in a 100–75% ethanol series (5 min each) and a distilled water wash. The tumor tissue sections were subsequently immersed in EDTA antigen repair buffer (pH 8.0), and the antigen was recovered by microwaving (10 min). We then added 3% BSA to block nonspecific binding sites for 30 min. The slides were subsequently incubated with the primary antibody rabbit anti-mouse CD4 (1:200, #GB13064-2, Servicebio) or the primary antibody rabbit anti-mouse CD8 (1:200, #GB11068, Servicebio) overnight at 4 °C. The tissue sections were then rinsed in PBS (pH 7.4) and incubated with FITC-conjugated goat anti-rabbit IgG (H + L) (1:100, #GB22303, Servicebio) at room temperature for 50 min in the dark. The sections were incubated in DAPI solution for 10 min at room temperature, and immunofluorescent images were taken using a Nikon Eclipse C1 microscope (Japan) and using Image J to evaluate the positive rates of CD4 and CD8 proteins.

### Statistical analysis

All statistical analyses were performed using SPSS 24.0 software (SPSS, IBM, Armonk, NY, USA) and GraphPad Prism V8.0 (GraphPad Software, CA, USA). All data were analyzed using a homogeneity of variance test and normal distribution test. Measurement data were expressed as the mean ± SD. Data comparisons between two groups were conducted using the unpaired t-test. Data among multiple groups were compared by one-way ANOVA followed by Tukey’s post hoc test. Repeated-measures ANOVA was used to compare data at different time points. We performed survival analysis using the Kaplan–Meier test and the log-rank method to compare Kaplan–Meier survival curves. All in vitro experiments were carried out at least in triplicate. *P* < 0.05 was considered statistically significant.

## Results

### High expression of LINC01123 and B7–H3 in HNSCC indicates poor prognosis, and miR-214-3P is the potential miRNA link between them

**Fig. 1:**
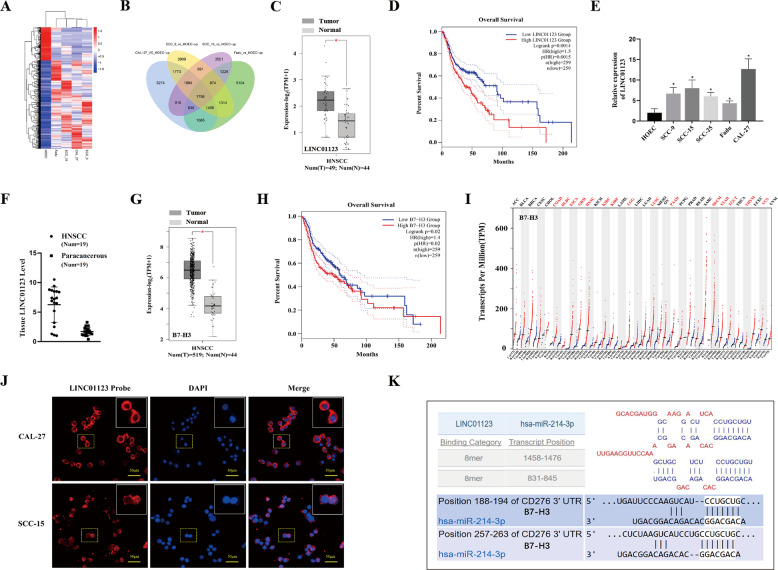
Elevated expression of LINC01123 and *B7–H3* in HNSCC is linked to poor prognosis, and the potential miRNA link between LINC01123 and *B7–H3* was predicted to be miR-214-3P via bioinformatic methods. **A** Heat map with hierarchical clustering. Blue and red indicate low and high expression levels, respectively. **B** Venn diagram of upregulated LncRNAs in four HNSCC cell lines (CAL-27, Fadu, SCC-9, SCC-15) compared with HOEC. **C** TCGA data of LINC01123 expression in the classical subtype HNSCC tissues (*n* = 49) and normal tissues (*n* = 44). **D** The overall survival rate analysis of LINC01123. **E** Expression of LINC01123 in HNSCC cell lines (SCC-9, SCC-15, SCC-25, Fadu, CAL-27) and HOEC. **F** Expression of LINC01123 in HNSCC patient tissues and paracancerous tissues, as detected by qRT-PCR. **G** TCGA data of *B7–H3* expression in HNSCC tissues (*n* = 519) and normal tissues (*n* = 44). **H** The overall survival rate analysis of *B7–H3*. **I** *B7–H3* is highly expressed in various types of tumor (COAD, DLBC, ESCA, GBM, KIRC, KIRP, LGG, LUSC, PAAD, SKCM, STAD, TGCT, THYM, and UCS). Each dot represents expression of samples. **J** Subcellular location of LINC01123, as detected by FISH assay. **K** Prediction of potential miRNA link between LINC01123 and *B7–H3*. COAD colon adenocarcinoma, DLBC lymphoid neoplasm diffuse large b-cell lymphoma, ESCA esophageal carcinoma, GBM glioblastoma multiforme, KIRC kidney renal clear cell carcinoma, KIRP kidney renal papillary cell carcinoma, LGG brain lower grade glioma, LUSC lung squamous cell carcinoma, PAAD pancreatic adenocarcinoma, SKCM skin cutaneous melanoma, STAD stomach adenocarcinoma, TGCT testicular germ cell tumors, THYM thymoma, UCS uterine carcinosarcoma. The experiment was repeated three times.

The heat maps show clusters of LncRNA with a similar pattern of expression that shares a common function or participates in a common signaling pathway (Fig. 1A). The Venn diagram shows 1756 overlapping LncRNA that were significantly upregulated in four HNSCC cell lines (CAL-27, Fadu, SCC-9, and SCC-15, *P* < 0.05) (Fig. 1B). Based on the data obtained from the TCGA public database on the GEPIA platform (http://gepia.cancer-pku.cn/) and our 1756 overlapping LncRNA datasets, we analyzed the LncRNA sequencing data of HNSCC. The results showed that LINC01123 was highly expressed in the classical subtype HNSCC (Fig. 1C), and its level of expression was significantly associated with poor patient prognosis (Fig. 1D). When total RNA was extracted from HNSCC cell lines (SCC-9, SCC-15, SCC-25, Fadu, and CAL-27) and human oral epithelial cells (HOEC) and the level of LINC01123 expression detected by qRT-PCR, high LINC01123 expression was seen in all HNSCC cell lines, among which the expression of LINC01123 was the highest in CAL-27. However, the expression level of LINC01123 in HOEC was low (Fig. 1E). The qRT-PCR results for the HNSCC tissue samples were consistent with those for the cell lines. Compared with paracancerous tissues, the level of LINC01123 expression in HNSCC tissues was high (Fig. 1F).

*B7–H3* was highly expressed in HNSCC (Fig. 1G), and its expression was associated with the prognosis of the disease (Fig. 1H). In addition, we obtained the following results through the GEPIA website, *B7–H3* was also expressed in multiple tumor types, e.g., colon adenocarcinoma, lymphoid neoplasm diffuse large B-cell lymphoma, esophageal carcinoma, glioblastoma multiforme, kidney renal clear cell carcinoma, kidney renal papillary-cell carcinoma, brain lower-grade glioma, lung squamous-cell carcinoma, pancreatic adenocarcinoma, skin cutaneous melanoma, stomach adenocarcinoma, testicular germ cell tumors, thymoma, and uterine carcinosarcoma (Fig. 1I).

Next, the subcellular location of LINC01123 was detected in CAL-27 and SCC-15 cell lines using the relevant FISH probes, and LINC01123 was mainly expressed in the cytoplasm and rarely detected in the nucleus (Fig. 1J). The subcellular localization of LINC01123 in SCC-9 and Fadu was shown in Fig. S1 in supplementary materials. Using the prediction websites LncBase and Targetscan (http://www.microrna.gr/LncBase, http://www.targetscan.org), we hypothesized that the miRNA link between LINC01123 and *B7–H3* is miR-214-3P (Fig. 1K).

### LINC01123 regulates B7–H3 by competitively binding to miR-214-3P

**Fig. 2:**
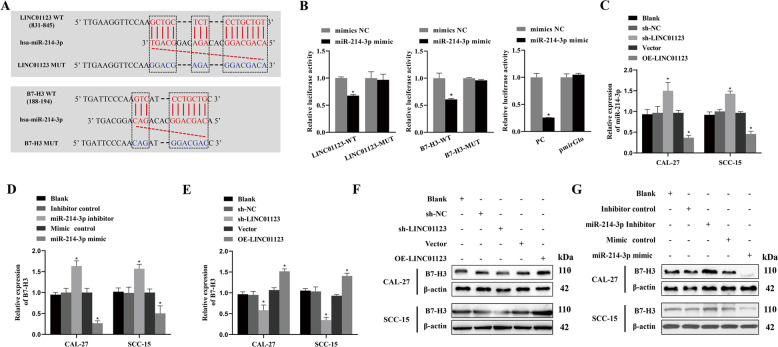
LINC01123 regulates the expression of *B7–H3* through sponging miR-214-3P. **A** Plasmids containing wild-type (WT) or mutant (MUT) 3′UTR of LINC01123 and *B7–H3* sequences were constructed. **B** Dual-luciferase-reporter gene assay was performed to evaluate the direct interactions between LINC01123, *B7–H3*, and miR-214-3P. **C** Effect of LINC01123 on miR-214-3P expression, as detected by qRT-PCR. **D** Effect of miR-214-3P on *B7–H3* expression, as detected by qRT-PCR. **E** Effect of LINC01123 on *B7–H3* expression, as detected by qRT-PCR. **F** Western blotting was used to detect the impact of LINC01123 on *B7–H3* expression. **G** Western blotting was used to detect the effects of miR-214-3P on *B7–H3* expression. *P < 0.05 compared with cells without treatment. WT wild-type sequences were constructed into the PmirGlo vector group, MUT mutant sequences were constructed into the PmirGlo vector group, PC positive control group, PmirGlo empty plasmid group, NC normal control, OE over-expression. The experiment was repeated three times.

To detect whether miR-214-3P binds to the predicted target sites in LINC01123 and *B7–H3*, we constructed WT and MUT luciferase-reporter vectors for LINC01123 and *B7–H3* (Fig. 2A), and a dual-luciferase reporter assay was conducted to verify the bioinformatic predictions (Fig. 2B). Compared with the NC-mimic group, the luciferase expression levels of the miR-214-3P mimic and LINC01123-WT plasmid cotransfection group were significantly decreased after 48 h (*P* < 0.05), but the luciferase activity of LINC01123-MUT was uninhibited. The assay also confirmed the predicted binding sites between the 3′UTR of *B7–H3* and miR-214-3P. Compared with the NC-mimic group, the luciferase expression levels of *B7–H3*-WT were significantly inhibited by transfection with the miR-214-3P mimics (*P* < 0.05), but the luciferase activity of *B7–H3*-MUT was not attenuated. The PC-group results showed that the experimental system was stable and reliable.

We used qRT-PCR to detect the effect of LINC01123 on miR-214-3P expression in HNSCC cells (Fig. 2C), and miR-214-3P expression was significantly upregulated in HNSCC cells when LINC01123 was silenced (*P* < 0.05). In addition, the overexpression of LINC01123 led to decreased miR-214-3P expression (*P* < 0.05), and a similar effect of miR-214-3P on *B7–H3* expression was detected by qRT-PCR (Fig. 2D). When miR-214-3P was inhibited, the expression of *B7–H3* increased significantly (*P* < 0.05), while the miR-214-3P mimic resulted in decreased expression of *B7–H3* (*P* < 0.05). When the effect of LINC01123 was detected by qRT-PCR, we found that LINC01123 silencing downregulated the expression of *B7–H3* (*P* < 0.05), while LINC01123 overexpression led to increased expression of *B7–H3* (*P* < 0.05) (Fig. 2E). The competitive binding of LINC01123 to miR-214-3P and *B7–H3* was further analyzed by western blot analysis. LINC01123 expression was positively correlated (Fig. 2F) and miR-214-3P expression was negatively correlated with *B7–H3* protein expression levels (Fig. 2G).

### Downregulation of LINC01123 or B7–H3 or upregulation of miR-214-3p inhibited HNSCC cell proliferation, migration, and invasion in vitro

**Fig. 3:**
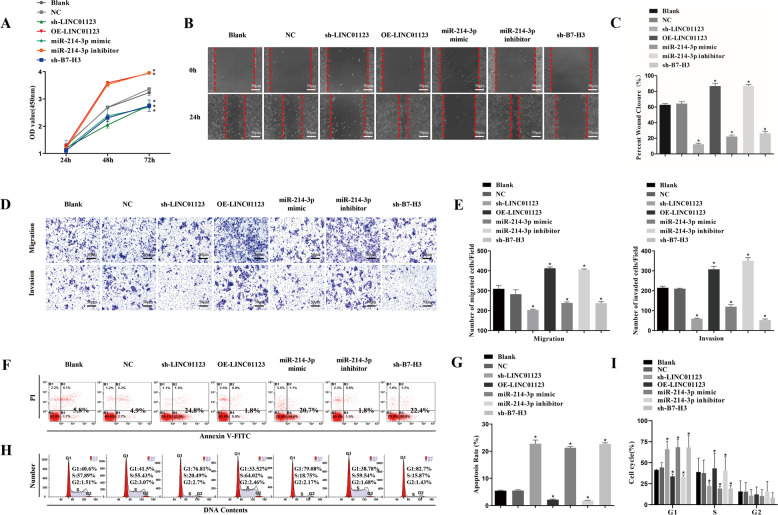
Downregulation of LINC01123 or *B7–H3* or upregulation of miR-214-3p inhibited HNSCC cell proliferation, migration, and invasion in vitro. HNSCC cells (CAL-27) were treated with sh-LINC01123, OE-LINC01123, miR-214-3p mimic, miR-214-3p inhibitor, or sh-*B7–H3*. **A** Cell Counting Kit 8 (CCK8) assay of the proliferation of HNSCC cells. **B** Motility of HNSCC cells was determined by scratch wound healing assay. **C** Statistical results of scratch wound healing assay. **D** Migration and invasion of HNSCC cells were determined by transwell migration and invasion assay. **E** Statistical analysis of transwell results. **F** Apoptosis rate of HNSCC cells, as detected by flow cytometry. **G** Statistical results of the apoptosis rate. **H** Cell cycle stages of HNSCC cells, as detected by flow cytometry. **I** Statistical analysis of the cell-cycle results. *P < 0.05 versus the blank group. The experiment was repeated three times.

We evaluated CAL-27 and SCC-15 cell proliferation by employing Cell Counting Kit 8 (CCK8) assays (Fig. 3A; Supplementary Fig. S2A), and found that downregulation of LINC01123 or *B7–H3* or upregulation of miR-214-3p significantly inhibited cell proliferation (*P* < 0.05), while LINC01123 overexpression or miR-214-3p inhibition led to the opposite (*P* < 0.05). The motility of HNSCC cells was then measured by scratch wound-healing assay (Fig. 3B, C; Supplementary Fig. S2C, I). LINC01123 overexpression or miR-214-3p downregulation increased the motility of CAL-27 and SCC-15 cells (*P* < 0.05). When LINC01123 or *B7–H3* expression was decreased or miR-214-3p expression was increased, the opposite results appeared (*P* < 0.05). Similarly, the transwell assay (Fig. 3D, E; Supplementary Fig. S2E, K, L) revealed that inhibition of LINC01123 or *B7–H3* or enhancement of miR-214-3p reduced tumor invasion and migration ability (*P* < 0.05), while the opposite was seen when LINC01123 was overexpressed or miR-214-3p was inhibited (*P* < 0.05).

The annexin-V FITC apoptosis assay (Fig. 3F, G; Supplementary Fig. S2G, N) showed that inhibition of LINC01123 or *B7–H3* or upregulation of miR-214-3p significantly increased the number of apoptotic cells (*P* < 0.05). In addition, the apoptotic rate of cells in the LINC01123 overexpression and miR-214-3p inhibition groups decreased (*P* < 0.05). When the cell-cycle distribution of CAL-27 cells was detected by flow cytometry (Fig. 3H, I), we observed an increase in the proportion of cells in the G1 phase and a decrease in the percentage of cells in the S phase in the sh-LINC01123, miR-214-3p mimic, and sh-*B7–H3* groups (*P* < 0.05). However, treatment of cells with OE-LINC01123 or the miR-214-3p inhibitor reduced the G1 phase and increased the S-phase cell proportions (*P* < 0.05). There were no significant differences in the percentages of cells in the G2 phase among the groups (*P* > 0.05). These results show that inhibition of LINC01123 or *B7–H3* or upregulation of miR-214-3p stalled the cell cycle at the G1 stage, leading to reduced cell proliferation.

### Upregulation of LINC01123 or B7–H3 or downregulation of miR-214-3p in HNSCC cells induces dysfunction of CD8^+^T cells

**Fig. 4:**
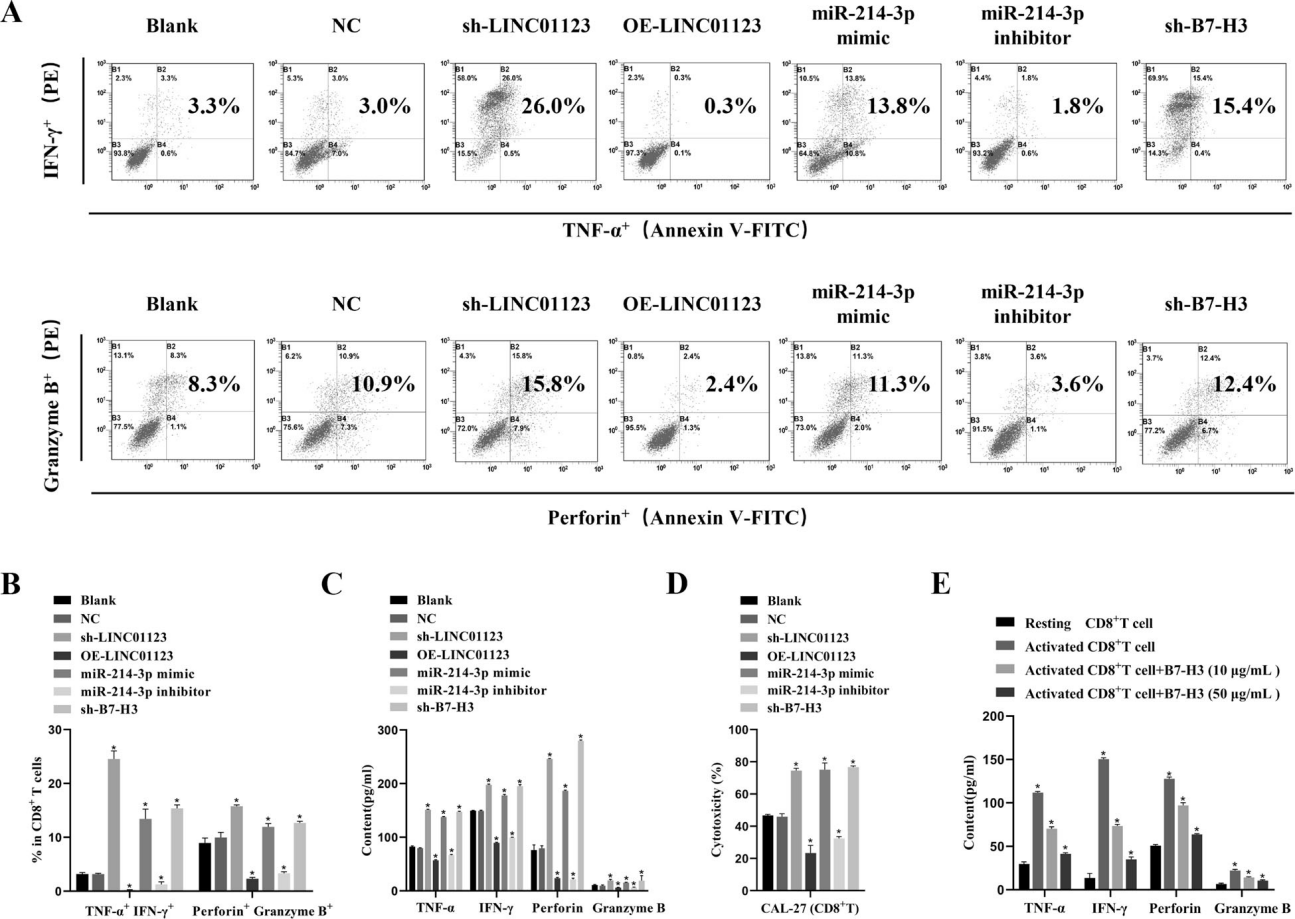
Upregulation of LINC01123 or *B7–H3* or downregulation of miR-214-3p induces dysfunction of CD8^+^T cells. CD8^+^T cells were isolated from PBMCs using immunomagnetic beads. After activation and amplification, CD8^+^T cells were cocultured with different CAL-27 cell treatment groups. **A** Expression of TNF-α, IFN-γ, perforin, and granzyme B in CD8^+^T-cell population was analyzed with flow cytometry. **B** Statistical analysis of the results from flow cytometry of CD8^+^T cells. **C** Expression levels of TNF-α, IFN-γ, perforin, and granzyme B in CAL-27–CD8^+^T cocultured supernatants, as detected by ELISA. **D** CCK8 cytotoxicity test of CD8^+^T cells. **E** ELISA was used to assess the immune activity of CD8^+^T cells after recombinant human *B7–H3* treatment. *P < 0.05 compared with cells without treatment. The experiment was repeated three times.

To investigate whether the expression of LINC01123, *B7–H3*, or miR-214-3p in HNSCC cells affects CD8^+^T-cell function, we analyzed the expression of TNF-α, IFN-γ, perforin, and granzyme B in the CD8^+^T cells by flow cytometry after coculture with the tumor cells (Fig. 4A, B). The positive rate of TNF-α^+^, IFN-γ^+^, CD8^+^T, and perforin^+^ granzyme B^+^ CD8^+^T cells increased after treatment with sh-LINC01123, sh-*B7–H3*, or the miR-214-3p mimic (*P* < 0.05). However, treatment with OE-LINC01123 or the miR-214-3p inhibitor induced the opposite result (*P* < 0.05).

The levels of TNF-α, IFN-γ, perforin, and granzyme B in CAL-27–CD8^+^T coculture supernatants were detected by ELISA (Fig. 4C). HNSCC cells transfected with sh-LINC01123, sh-*B7–H3*, or the miR-214-3p mimic exhibited higher levels of TNF-α, IFN-γ, perforin, and granzyme B (*P* < 0.05), and cells treated with OE-LINC01123 or the miR-214-3p inhibitor led to the opposite result (*P* < 0.05). Furthermore, the ELISA results showed that CD8^+^T cells treated with recombinant human *B7–H3* exhibited lower levels of TNF-α, IFN-γ, perforin, and granzyme B (*P* < 0.05), and as the concentration of *B7–H3* increased, the amount of activity factors secreted by CD8^+^T cells decreased (Fig. 4E).

In addition, the CCK8 assay (Fig. 4D) showed that silencing LINC01123 or *B7–H3* expression in CAL-27 cells or miR-214-3p-mimic treatment of CAL-27 cells enhanced the tumor-cell-killing effect of CD8^+^T cells (*P* < 0.05), while LINC01123 overexpression or miR-214-3p inhibitor led to the opposite (*P* < 0.05). Therefore, according to the above results, LINC01123 or *B7–H3* overexpression or miR-214-3p inhibitor in HNSCC cells induced the dysfunction of CD8^+^T cells.

### Downregulation of LINC01123 or B7–H3 or upregulation of miR-214-3p inhibits HNSCC cell tumorigenicity and promotes the secretion of immune-related factors in vivo

**Fig. 5:**
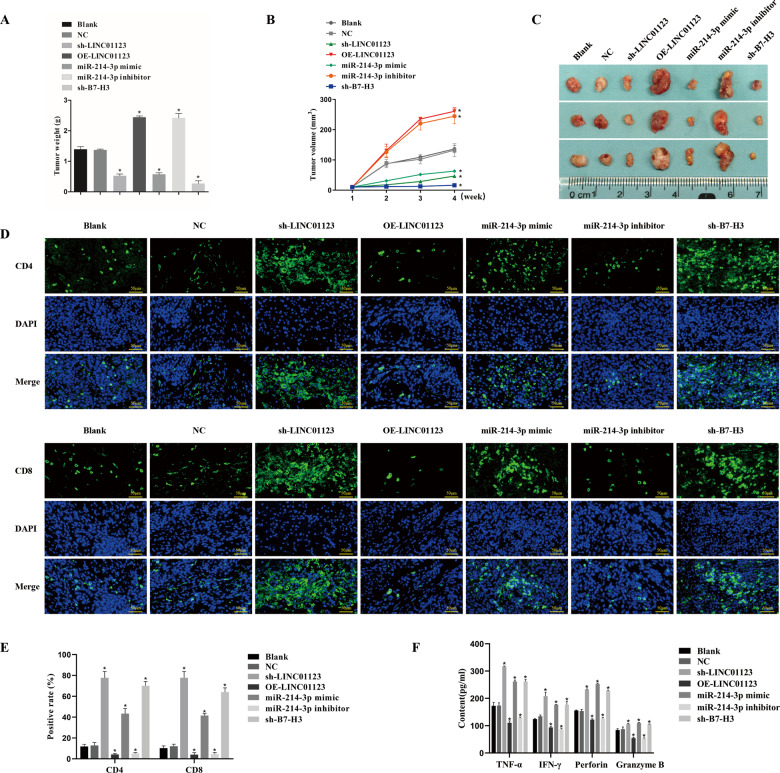
Downregulation of LINC01123 or *B7–H3* or upregulation of miR-214-3p inhibits the tumorigenicity and immune escape of HNSCC cells in vivo. SCC-7 cells were subcutaneously injected into C3H mice and treated with sh-LINC01123, OE-LINC01123, miR-214-3p mimic, miR-214-3p inhibitor, or sh-*B7–H3* after tumor formation. **A** Tumor weights of mice from each group. **B** Tumor-volume growth curve of each group (*n* = 3). **C** Tumor images at week 4. **D** Immunofluorescence staining of CD4 and CD8 in tumor specimens (×400). **E** Statistical analysis of CD4 and CD8 immunofluorescence results. **F** Expression of TNF-α, IFN-γ, perforin, and granzyme B in peripheral blood of tumor-bearing C3H mice, as detected by ELISA. *P < 0.05 versus the blank group. The experiment was repeated three times.

To examine the effects of LINC01123, *B7–H3*, and miR-214-3p on tumorigenicity in vivo, SCC-7 cells were subcutaneously injected into C3H mice to establish a cell-derived xenograft model. The weight and volume (Fig. 5A–C) of tumors were significantly inhibited in C3H mice after treatment with sh-LINC01123, sh-*B7–H3*, or the miR-214-3p mimic (*P* < 0.05), and they were increased after treatment with OE-LINC01123 or the miR-214-3p inhibitor (*P* < 0.05). In addition, the immunofluorescence assay (Fig. 5D, E) showed that tumor tissues treated with sh-LINC01123, sh-*B7–H3*, or the miR-214-3p mimic exhibited increased positive rates of CD4 and CD8 proteins (*P* < 0.05), and tumor tissues from mice treated with OE-LINC01123 or the miR-214-3p inhibitor had the opposite effect (*P* < 0.05).

Following this, the expression of TNF-α, IFN-γ, perforin, and granzyme B in the peripheral blood of tumor-bearing C3H mice was detected by ELISA (Fig. 5F). The peripheral blood of mice treated with sh-LINC01123, sh-*B7–H3*, or the miR-214-3p mimic exhibited higher levels of TNF-α, IFN-γ, perforin, and granzyme B (*P* < 0.05), and treatment of the mice with OE-LINC01123 or the miR-214-3p inhibitor led to the opposite finding (*P* < 0.05). Therefore, we concluded that the downregulation of LINC01123 or *B7–H3* or the overexpression of miR-214-3p inhibited the tumorigenicity of HNSCC cells and promoted the secretion of immune-related factors in vivo.

### miR-214-3p silencing reverses the function of LINC01123 in HNSCC

**Fig. 6:**
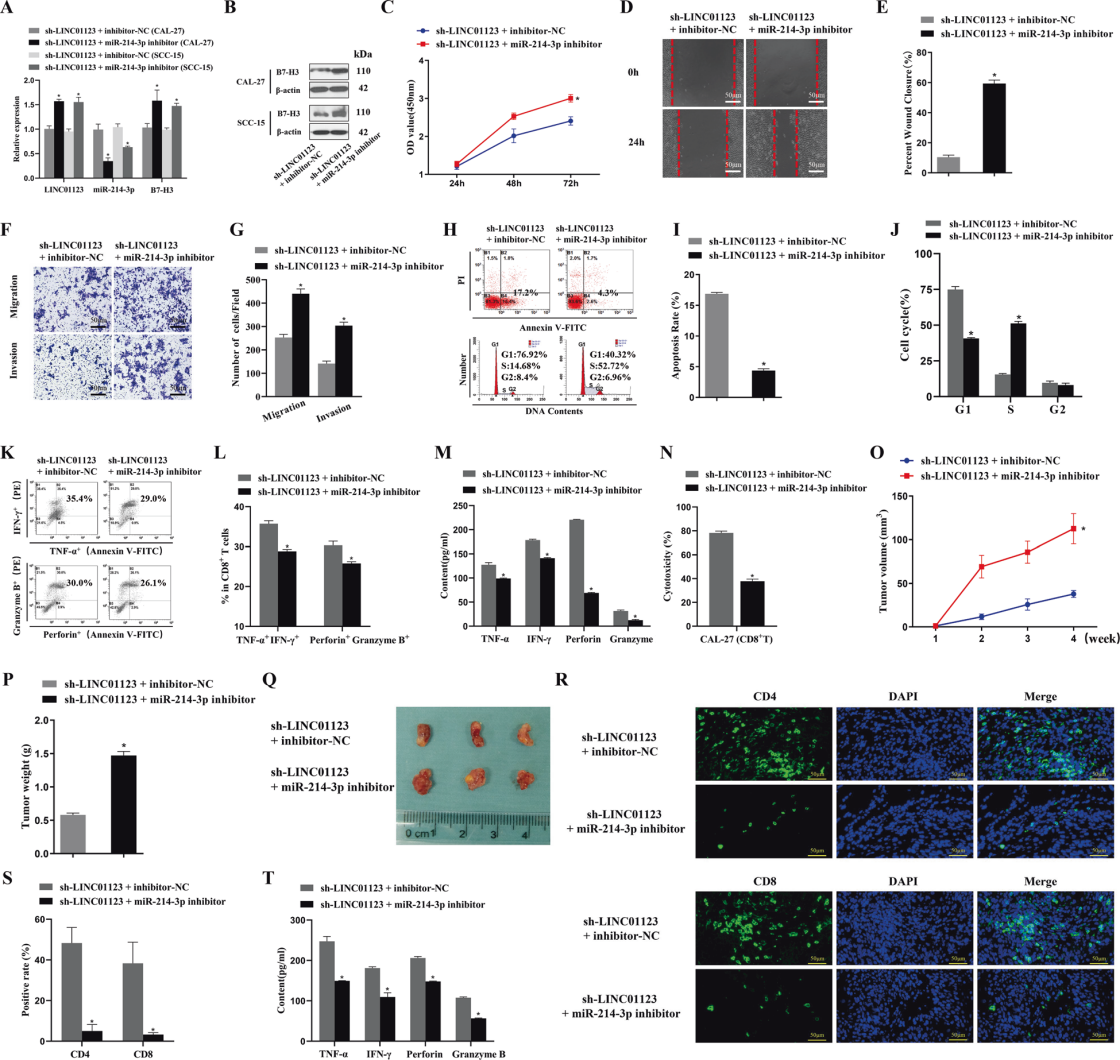
The function of LINC01123 in HNSCC can be reversed by miR-214-3p silencing. HNSCC cells were transfected with sh-LINC01123 in the presence of inhibitor–NC or miR-214-3p inhibitor. **A** Relative expression levels of LINC01123, miR-214-3p, and *B7–H3* were determined by qRT-PCR. **B** *B7–H3* protein expression was determined by western blot analysis. **C** CAL-27 cell viability was measured using a CCK8 assay. **D** Migration of CAL-27 cells was determined by scratch wound-healing assay. **E** Statistical results of the scratch-healing test. **F** Migration and invasion of CAL-27 cells, as determined by transwell migration and invasion assay. **G** Number of migration and invasion cells. **H** Apoptosis rate and cell-cycle stage were detected by flow cytometry. **I** Statistical analysis of apoptosis results. **J** Statistical analysis of cell cycle stage results. **K** CD8^+^T cells were cocultured with CAL-27 cells that had been transfected with plasmids, and the expression of TNF-α, IFN-γ, perforin, and granzyme B in the CD8^+^T cells was analyzed by flow cytometry. **L** Statistical analysis of CD8^+^T-cell flow cytometry results. **M** After different treatments, CAL-27 cells were cocultured with CD8^+^T cells, and the expression levels of TNF-α, IFN-γ, perforin, and granzyme B in coculture supernatants were detected by ELISA. **N** Killing effect of CD8^+^T cells on CAL-27 cells, as assessed by CCK8. **O** Tumor-volume growth curves of the sh-LINC01123 + inhibitor–NC group and sh-LINC01123 + miR-214-3p-inhibitor group. **P** Tumor weights of tumor-bearing C3H mice. **Q** Tumor images at week 4. **R** Immunofluorescence staining for CD4 and CD8 in tumor specimens (×400). **S** Statistical analysis of CD4 and CD8 immunofluorescence results. **T**. Expression of TNF-α, IFN-γ, perforin, and granzyme B in peripheral blood of tumor-bearing C3H mice, as detected by ELISA. *P < 0.05 versus the sh-LINC01123 + inhibitor–NC group. The experiment was repeated three times.

The sh-LINC01123 + miR-214-3p-inhibitor group exhibited upregulated LINC01123 and *B7–H3* expression, decreased miR-214-3p expression (Fig. 6A, B), elevated proliferation ability (Fig. 6C), increased cell motility (Fig. 6D, E) and invasion and migration ability (Fig. 6F, G), a reduced cell-apoptosis rate (Fig. 6H, I), a decreased proportion of cells in the G1 phase, and an increased proportion of cells in the S phase (Fig. 6H, J) compared with the sh-LINC01123 + inhibitor–NC group (*P* < 0.05). Similarly, SCC-15 cells in the sh-LINC01123 + miR-214-3p-inhibitor group had the same proliferation ability (Supplementary Fig. S2B), cell motility (Supplementary Fig. S2D, J), invasion and migration ability (Supplementary Fig. S2F, M), and apoptosis rate (Supplementary Fig. S2H, O) as CAL-27 cell line. In addition, the proportion of TNF-α^+^, IFN-γ^+^, CD8^+^T, and perforin^+^ granzyme B^+^ CD8^+^T cells decreased after treatment with sh-LINC01123 + miR-214-3p-inhibitor (*P* < 0.05) (Fig. 6K, L). In the ELISA of the sh-LINC01123 + miR-214-3p inhibitor group, lower levels of TNF-α, IFN-γ, perforin, and granzyme B were seen in the CAL-27–CD8^+^T cocultured supernatant (*P* < 0.05) (Fig. 6M). Furthermore, the CCK8 assay (Fig. 6N) showed the reduced killing effect of CD8^+^T cells on CAL-27 cells in the sh-LINC01123 + miR-214-3p-inhibitor group (*P* < 0.05).

The in vivo experiment revealed that the volume and weight (Fig. 6O–Q) of tumors were significantly increased in the sh-LINC01123 + miR-214-3p inhibitor group (*P* < 0.05). Immunofluorescence assays (Fig. 6R, S) showed that tumor tissues from mice treated with sh-LINC01123 + miR-214-3p-inhibitor exhibited decreased positive rates of CD4 and CD8 proteins (*P* < 0.05). Furthermore, there was downregulated expression of TNF-α, IFN-γ, perforin, and granzyme B in the peripheral blood of tumor-bearing C3H mice in the sh-LINC01123 + miR-214-3p-inhibitor group (*P* < 0.05) (Fig. 6T). The above results provide in vitro and in vivo evidence that the function of LINC01123 can be reversed by miR-214-3p silencing in HNSCC.

## Discussion

HNSCC is an immunosuppressive malignant tumor with high malignancy [26], and immunotherapy is an innovative treatment method with a broad clinical application prospect [27]. However, the effectiveness of immunotherapy is poor due to the variety of ways tumors can evade immune surveillance [28]. Therefore, exploring the immune-escape mechanism of tumor cells is expected to improve the efficacy of HNSCC immunotherapy. Recent studies have shown that several LncRNAs are associated with immune escape [29]. For example, LncRNA–MX1–215 negatively regulates immunosuppression by interrupting H3K27 acetylation in HNSCC [30]. In colorectal cancer, LncRNA–MIR17HG can upregulate the expression of PD-L1 by “sponging” with miR-17-5p, indicating its potential role in immunotherapy [31]. All the above studies indicated that LncRNAs play crucial roles in tumor immune escape.

**Fig. 7:**
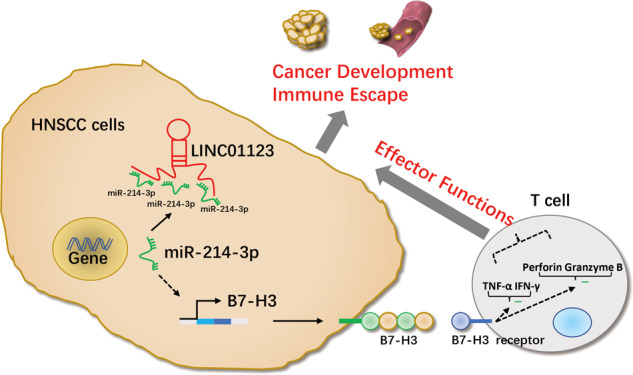
Molecular role of LINC01123 in regulating the progression and immune escape of HNSCC.

Initially, we analyzed 1756 overlapping LncRNA datasets and found that the expression of LINC01123 was significantly elevated (*P* < 0.05). We then analyzed the LncRNA sequencing data for HNSCC in the TCGA database [32] and found that high LINC01123 expression was linked to inferior patient prognoses. However, it was confusing to find that LINC01123 expression in HNSCC tissues was not statistically different from that in normal tissues in the TCGA database. During a literature review, we found that the gene expression subtypes of HNSCC can be divided into four types: atypical (24%), mesenchymal (27%), basal (31%), and classical (18%). Furthermore, studies [33] have revealed that the classical HNSCC subtype is highly correlated with the mutation of TP53, CDKN2A loss of function, and changes to oxidative-stress genes, indicating that the subtype is highly correlated with tumorigenesis and progression. Therefore, LncRNA sequencing data for the classical subtype HNSCC were selected from the TCGA database for analysis. Surprisingly, LINC01123 expression in the classical subtype HNSCC was significantly higher (*P* < 0.05) than that of normal tissues. To verify the findings, we detected the expression of LINC01123 in HNSCC cell lines and tissues using qRT-PCR, and the results confirmed that the LncRNA was highly expressed in HNSCC cell lines (SCC-9, SCC-15, SCC-25, Fadu, and CAL-27) and tumor specimens.

*B7–H3* is highly expressed in a variety of tumors, e.g., esophageal carcinoma [34] and kidney renal clear cell carcinoma [35], etc. Research has already confirmed that *B7–H3* is involved in the immune escape mechanism of HNSCC [20], and the *B7–H3* checkpoint may serve as a novel target for cancer immunotherapy [19]. However, its specific mechanism of regulation is still unclear. Our results indicated that *B7–H3* was highly expressed in HNSCC, and its high expression was associated with a poor prognosis. Using bioinformatics, we found and confirmed that the miRNA link between LINC01123 and *B7–H3* is miR-214-3P. Many studies have shown that miR-214-3p is abnormally expressed in a variety of malignant tumors and is significantly associated with the growth, metastasis, recurrence, and poor prognosis of malignant tumors [22, 36]. Our results confirmed that LINC01123 and *B7–H3* were highly expressed in HNSCC cells, whereas miR-214-3P showed reduced expression. In addition, our study showed that LINC01123 and *B7–H3* were competitively bound to miR-214-3P in HNSCC cells.

In subsequent assays, we found that downregulation of LINC01123 and *B7–H3* or upregulation of miR-214-3p reduced HNSCC tumor progression. It is worth noting that *B7–H3* is not only associated with tumor immune escape but also promotes tumor-cell invasion through a nonimmune pathway. Furthermore, overexpression of LINC01123 or *B7–H3* or downregulation of miR-214-3p in HNSCC cells induced dysfunction of CD8^+^T cells. Notably, the expression level of *B7–H3* was negatively correlated with the expression of CD8^+^T-cell activity factors TNF-α, IFN-γ, perforin, and granzyme B, which could explain why high LINC01123 expression promotes tumor immune escape. Finally, we confirmed that the function of LINC01123 could be reversed by miR-214-3p silencing in HNSCC.

In conclusion, our study suggested that LINC01123 promotes the progression of HNSCC (Fig. 7). The overexpression of LINC01123 inhibited CD8^+^T-mediated immune escape by HNSCC cells by upregulating *B7–H3* through sponging miR-214-3p. Targeted blocking of LINC01123 may improve anti-tumor immunity; reverse CD8^+^T-cell dysfunction; and ultimately, limit tumor proliferation, metastasis, and recurrence; and improve the prognosis of patients. However, the receptor for *B7–H3* has not been determined [37], and its functions and mechanisms in relation to immune escape still need to be elucidated. Moreover, the focus of future studies should be the mechanisms involved in controlling the exosome-mediated immune microenvironment by tumor cells [38].

## Supplementary information

Supplemental Material

Supplemental Material Figure S1

Supplemental Material Figure S2

aj-checklist

author-contribution-form

## Data Availability

The datasets used or/and analyzed during the current study are available from the corresponding author on reasonable request.
