# Supplementary material for: LINC01123 promotes immune escape by sponging miR-214-3p to regulate B7–H3 in head and neck squamous-cell carcinoma
Source: Cell Death Dis. 2022 Feb 3;13(2):109. doi: 10.1038/s41419-022-04542-0 (PMC8814033; doi:10.1038/s41419-022-04542-0)
Supplement: Supplementary file 1 — Supplemental Material [file 41419_2022_4542_MOESM1_ESM.docx]

## Supplementary Figures

Figure S1:

Subcellular localization of LINC01123 in SCC-9 and Fadu were detected by FISH.

Figure S2:

HNSCC cells (SCC-15) were treated with sh-LINC01123, OE-LINC01123, miR-214-3p mimic, miR-214-3p inhibitor, sh-*B7-H3*, sh-LINC01123 + inhibitor-NC, sh-LINC01123 + miR-214-3p inhibitor. A, B) CCK8 assay of the proliferation of SCC-15 cells. C, D) Motility of SCC-15 cells was determined by scratch wound healing assay. E, F) Migration and invasion of SCC-15 cells were determined by transwell migration and invasion assay. G, H) Apoptosis rate of SCC-15 cells, as detected by flow cytometry.

I, J) Statistical results of scratch wound healing assay. K, L, M) Statistical analysis of transwell results. N, O) Statistical results of the apoptosis rate. **P* < 0.05 versus the blank group and the sh-LINC01123 + inhibitor-NC group.

## Supplementary Table

Table S1**:** The human population, sample size, clinical characteristics, and HPV status information

| **Clinical data** | **HNSCC patients (n=19)** | |
| --- | --- | --- |
|  | n | % |
| **Age, years** |  |  |
| >60 | 11 | 57.9 |
| <=60 | 8 | 42.1 |
| **Gender** |  |  |
| Male | 9 | 47.4 |
| Female | 10 | 52.6 |
| **T** |  |  |
| T1-2 | 5 | 26.3 |
| T3-4 | 14 | 73.7 |
| **M** |  |  |
| M0 | 18 | 94.7 |
| M1 | 1 | 5.30 |
| **N** |  |  |
| N0 | 8 | 42.1 |
| N1-3 | 11 | 57.9 |
| **HPV** |  |  |
| + | 3 | 15.8 |
| - | 16 | 84.2 |
| **Perineural invasion** |  |  |
| YES | 6 | 31.6 |
| NO | 13 | 68.4 |

T tumor status, N regional lymph nodes status, M metastasis status
