# Supplementary figures and images for: LINC01123 promotes immune escape by sponging miR-214-3p to regulate B7–H3 in head and neck squamous-cell carcinoma
Source: Cell Death Dis. 2022 Feb 3;13(2):109. doi: 10.1038/s41419-022-04542-0 (PMC8814033; doi:10.1038/s41419-022-04542-0)

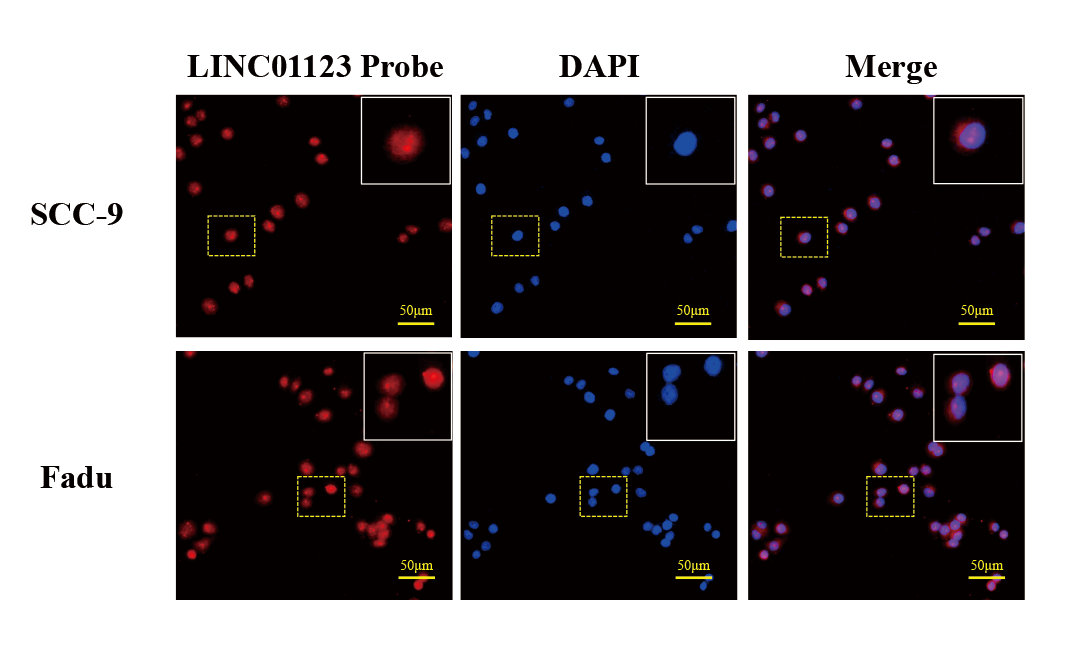

Supplement: Supplementary file 2 — Supplemental Material Figure S1 [file 41419_2022_4542_MOESM2_ESM.png]

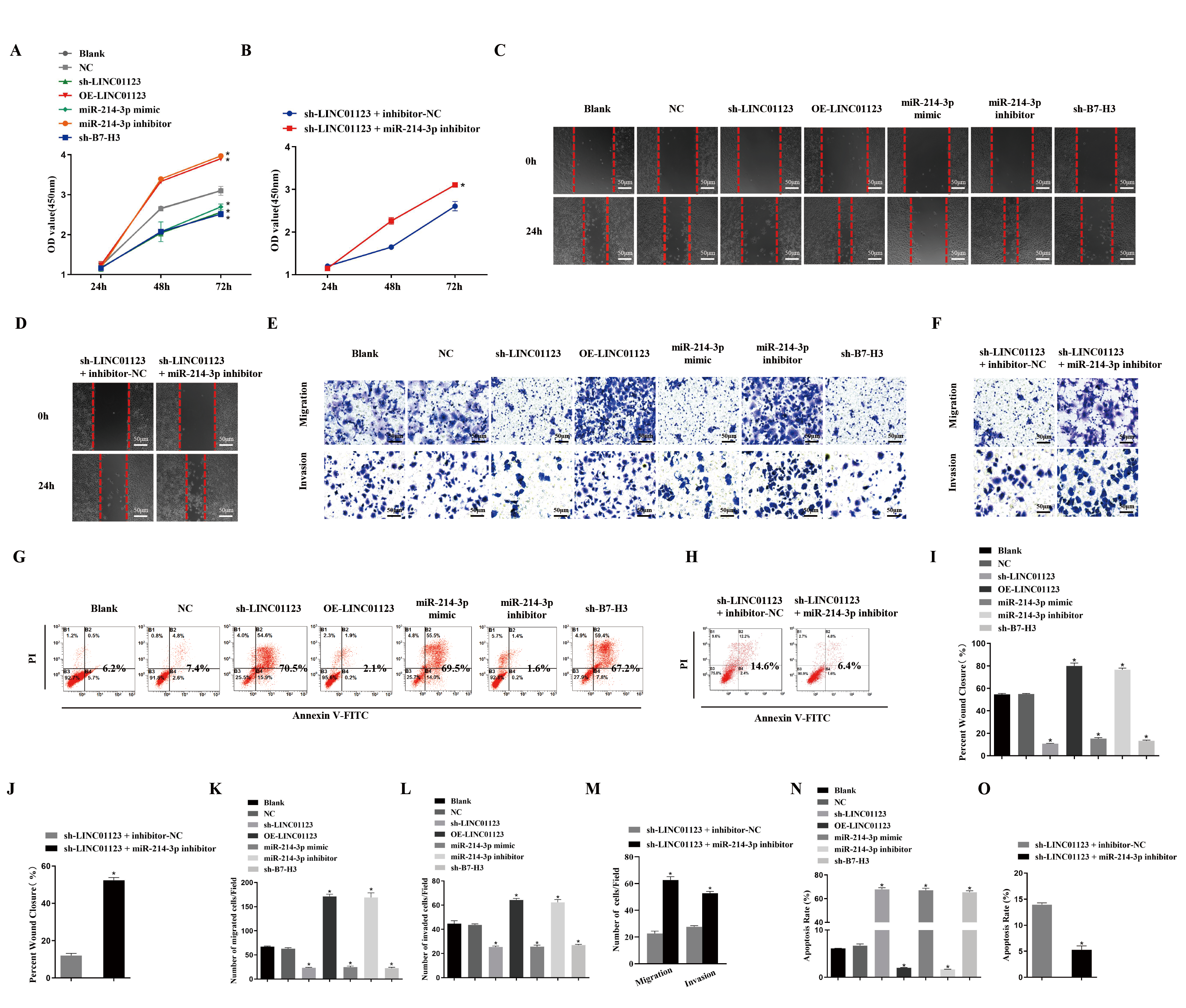

Supplement: Supplementary file 3 — Supplemental Material Figure S2 [file 41419_2022_4542_MOESM3_ESM.png]
