# Supplementary material for: LINC01123 promotes immune escape by sponging miR-214-3p to regulate B7–H3 in head and neck squamous-cell carcinoma
Source: Cell Death Dis. 2022 Feb 3;13(2):109. doi: 10.1038/s41419-022-04542-0 (PMC8814033; doi:10.1038/s41419-022-04542-0)
Supplement: Supplementary file 5 — author-contribution-form [file 41419_2022_4542_MOESM5_ESM.pdf]

DECLARATION OF CONTRIBUTIONS TO ARTICLE

ADMC

Manuscript Number: CDDIS-21-2092

Journal Name: *Cell Death & Disease* (the 'Journal')

Proposed Title of the Contribution:

LINC01123 promotes immune escape by sponging miR-214-3p to regulate B7-H3 in head and neck squamous cell carcinoma (the 'Contribution)

Author(s):

Huan Li 1, *, Zihui Yang 1, *, Xiangming Yang 1, *, Fengrui Zhang 1, Jun Wang 1, Zhongming Wu 1, Chaojie Wanyan 1, Qingzhe Meng 1, Wanpeng Gao 1, Xinjie Yang 1, #, Jianhua Wei 1, # (the 'Authors')

For all *CDDis* articles, each person named as an author in the published version must be able to show he or she has contributed substantially to the article.

Authorship credit should be based on 1) substantial contributions to conception and design, acquisition of data, or analysis and interpretation of data; 2) drafting the article or revising it critically for important intellectual content; and 3) final approval of the version to be published. Authors should meet conditions 1, 2 and 3.

Any person who cannot be shown to have made a substantial contribution to the article cannot be listed as an author in the final version. The name of any person who is deemed to have made a minor contribution can, however, appear in the Acknowledgments section of the article.

Please complete the table below to indicate the contributions of all named authors to the manuscript.

Author Full Name:	Specification of Contribution to the Manuscript:
Huan Li	1) substantial contributions to conception and design, acquisition of data, or analysis and interpretation of data; 2) drafting the article or revising it critically for important intellectual content; and 3) final approval of the version to be published.
Zihui Yang	1) substantial contributions to conception and design, acquisition of data, or analysis and interpretation of data; 2) drafting the article or revising it critically for important intellectual content; and 3) final approval of the version to be published.
Xiangming Yang	1) substantial contributions to conception and design, acquisition of data, or analysis and interpretation of data; 2) drafting the article or revising it critically for important intellectual content; and 3) final approval of the version to be published.
Fengrui Zhang	1) substantial contributions to conception and design, acquisition of data, or analysis and interpretation of data; 2) drafting the article or revising it critically for important intellectual content; and 3) final approval of the version to be published.
Jun Wang	1) substantial contributions to conception and design, acquisition of data, or analysis and interpretation of data; 2) drafting the article or revising it critically for important intellectual content; and 3) final approval of the version to be published.
Zhongming Wu	1) substantial contributions to conception and design, acquisition of data, or analysis and interpretation of data; 2) drafting the article or revising it critically for important intellectual content; and 3) final approval of the version to be published.
Chaojie Wanyan	1) substantial contributions to conception and design, acquisition of data, or analysis and interpretation of data; 2) drafting the article or revising it critically for important intellectual content; and 3) final approval of the version to be published.
Qingzhe Meng	1) substantial contributions to conception and design, acquisition of data, or analysis and interpretation of data; 2) drafting the article or revising it critically for important intellectual content; and 3) final approval of the version to be published.
Wanpeng Gao	1) substantial contributions to conception and design, acquisition of data, or analysis and interpretation of data; 2) drafting the article or revising it critically for important intellectual content; and 3) final approval of the version to be published.
Xinjie Yang	1) substantial contributions to conception and design, acquisition of data, or analysis and interpretation of data; 2) drafting the article or revising it critically for important intellectual content; and 3) final approval of the version to be published.
Jianhua Wei	1) substantial contributions to conception and design, acquisition of data, or analysis and interpretation of data; 2) drafting the article or revising it critically for important intellectual content; and 3) final approval of the version to be published.

Please complete the table below to indicate the contributions of all named authors to the figures.

Figure 1:

All authors participated in the production, statistical analysis and review of the charts

Figure 2:

All authors participated in the production, statistical analysis and review of the charts

Figure 3:

All authors participated in the production, statistical analysis and review of the charts

Figure 4:

All authors participated in the production, statistical analysis and review of the charts

Figure 5:

All authors participated in the production, statistical analysis and review of the charts

Figure 6:

All authors participated in the production, statistical analysis and review of the charts

Signed for and on behalf of the Author(s):	Print Name:	Date:
Huan Li	Huan Li	2021-9-25
